# Visualising varnish removal for conservation of paintings by fluorescence lifetime imaging (FLIM)

**DOI:** 10.1186/s40494-023-00957-w

**Published:** 2023-06-16

**Authors:** Christine B. Wilda, Aviva Burnstock, Klaus Suhling, Francesco Mattioli Della Rocca, Robert K. Henderson, Jakub Nedbal

**Affiliations:** 1 Department of Physics, King’s College London, Strand, London, WC2R 2LS United Kingdom; 2 The Courtauld, Somerset House, Strand, London, WC1X 0RN United Kingdom; 3 ConservArt, 6620 E Rogers Cir, Boca Raton, FL 33487 United States; 4 School of Engineering, University of Edinburgh, King’s Buildings, Edinburgh, EH9 3JL United Kingdom; 5 Europe Technology Development Centre, Sony Semiconductor Solutions - Sony Europe B.V., Trento, Italy

**Keywords:** Varnish removal, Painting conservation, Fluorescence lifetime, Time-correlated single photon counting (TCSPC), Single-photon avalanche diode (SPAD), Fluorescence lifetime imaging (FLIM), Time-resolved fluorescence spectroscopy

## Abstract

The removal of varnish from the surface is a key step in painting conservation. Varnish removal is traditionally monitored by examining the painting surface under ultraviolet illumination. We show here that by imaging the fluorescence lifetime instead, much better contrast, sensitivity, and specificity can be achieved. For this purpose, we developed a lightweight (4.8 kg) portable instrument for macroscopic fluorescence lifetime imaging (FLIM). It is based on a time-correlated single-photon avalanche diode (SPAD) camera to acquire the FLIM images and a pulsed 440 nm diode laser to excite the varnish fluorescence. A historical model painting was examined to demonstrate the capabilities of the system. We found that the FLIM images provided information on the distribution of the varnish on the painting surface with greater sensitivity, specificity, and contrast compared to the traditional ultraviolet illumination photography. The distribution of the varnish and other painting materials was assessed using FLIM during and after varnish removal with different solvent application methods. Monitoring of the varnish removal process between successive solvent applications by a swab revealed an evolving image contrast as a function of the cleaning progress. FLIM of dammar and mastic resin varnishes identified characteristic changes to their fluorescence lifetimes depending on their ageing conditions. Thus, FLIM has a potential to become a powerful and versatile tool to visualise varnish removal from paintings.

**Graphical Abstract:**

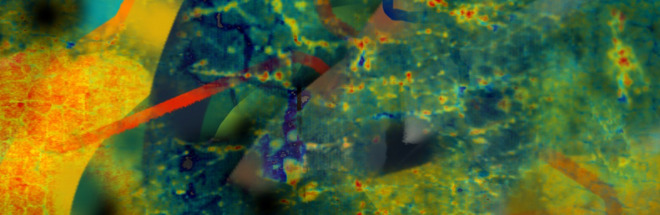

## Introduction

Conservation of paintings often involves the removal of varnish layers from the surface of paintings. The process includes a series of local varnish removal treatments to determine the most effective method. The progress of varnish removal is traditionally monitored by examining the painting surface under ultraviolet (UV) illumination if the varnish emits sufficiently bright UV-excited fluorescence. Conservation diagnostics is typically carried out by testing a range of solvents or reagents for varnish removal from different areas of a painting prior to the selective removal of one or more layers. This testing phase is critical to evaluate the effectiveness of the varnish removal. It requires a close inspection of the surface to examine the relationship between the paint and the varnish, and the presence of residual varnish that may be found in the interstices of the textured paint. Current methods for evaluating varnish removal and monitoring its removal use UV-excited fluorescence imaging that lacks the desirable contrast and specificity. Inspection of the UV-induced fluorescence emission from the varnish is based on fluorescence intensity and this depends on the type, age and thickness of the varnish. The paint and other painting materials can contribute to the emission, too. Moreover, the fluorescence light can be scattered, absorbed or reflected by the artwork, further complicating the overall fluorescence signature.

In addition to emission intensity, fluorescence can also be characterised by its excitation and emission spectra, fluorescence lifetime, and polarisation. By employing spectral or fluorescence lifetime measurements, a more specific discrimination of the varnish fluorescence from the fluorescence of other materials in the painting becomes possible [1]. Importantly, the fluorescence lifetime is independent of the amount of the varnish and is mostly insensitive to scattering and reabsorption.

There are a number of ways to measure fluorescence lifetimes and to perform fluorescence lifetime imaging (FLIM) [2–6]. Camera-based wide-field time-correlated single photon counting (TCSPC) [7–9] is particularly advantageous for this purpose, because (i) an image is obtained without scanning and (ii) TCSPC is the gold standard for fluorescence lifetime measurements. It has numerous advantages over other methods, for example single-photon sensitivity (thus allowing a low excitation light intensity), a high signal-to-noise ratio, a well-defined experimental uncertainty due to Poisson photon statistics, easy visualisation of fluorescence decays, and its capability to resolve multi-exponential fluorescence decays [10], which is relevant for the work presented here.

Fluorescence lifetime studies on artworks have previously demonstrated the ability of the technique to distinguish different art materials and their physical properties intractable by conventional UV-excited fluorescence imaging. For example, non-imaging, spectrally sensitive, and time-resolved fluorescence detection was used to identify pigments in a historical book [11]. A spectral and fluorescence lifetime analysis of a wall painting confirmed the presence and distribution of specific pigments and otherwise unnoticeable decorative features [12]. Fluorescence lifetime imaging of a fresco revealed the localisation of material introduced during historical conservation efforts [13]. Organic paint media were studied by fluorescence lifetime using two different excitation wavelengths and spectrally-resolved spectroscopy with four different excitation wavelengths to find that fluorescence lifetime had a greater specificity than spectral analysis, when used with violet (405 nm) excitation [14]. Short-lived fluorescence of organic pigments was discriminated by time-gated FLIM and spectral separation from long-lived semiconductor trapped-state fluorescence in historical paintings [15]. FLIM of marble statues revealed organic contamination on the surface not present in the control quarry marble sample [16] and a discolouration due to substances inside the stone and not on its surface [17]. Two studies using fluorescence lifetime measurements specifically focused on the investigation of varnish coatings. Spectrally-resolved time-gated fluorescence lifetime spectroscopy was performed on varnishes coating historical musical instruments in the search for a non-invasive method of discrimination between their different classes [18]. Fluorescence lifetime imaging and spectral analysis were performed on areas of a painting with a historical varnish and areas where it was removed with different solvents [19].

The aforementioned research on fluorescence lifetime analysis of artworks relied on time-gating of image-intensified cameras to measure the fluorescence decays. The gate widths were in the 3 ns–10 ns range and had jitter in excess of 0.5 ns [12–16, 18, 19]. The advantages of the time gating approach, compared to TCSPC, included its relatively simple implementation, earlier adoption and wider availability of suitable time-gated imaging detectors with a higher number of available pixels, which enabled larger fields of views. The limitations of the time-gating method included its lower photon efficiency due to the loss of all fluorescent photons outside the variable time gates, the higher jitter, and the wide time gates, which made accurate short (sub-nanosecond) lifetime determination and multi-exponential analysis difficult if not impractical.

In contrast, TCSPC offers a more accurate and photon efficient method to measure fluorescence lifetime and is suited to multi-exponential fluorescence decay analysis [20]. The only example of a report on multi-exponential fluorescence decay analysis of paintings, known to us, demonstrated the use of two-exponential fits on single points on the surface of a painting to characterise different paint pigments [11]. Later research using TCSPC to study painting materials did not exploit the opportunity to perform multi-exponential analysis on the data. It instead relied on non-fitting phasor approach for data analysis [21] or automated segmentation [22]. These studies analysed various painting materials and their combinations using a non-imaging TCSPC setup combined with Raman spectroscopy to find the spectroscopic signatures of these materials [21]. Besides TCSPC, a streak camera is another tool enabling more accurate fluorescence lifetime measurements compared to the widely used time-gating. It was used in parallel with the emission spectrum measurements in a non-imaging setup to characterise common pigments and resins [23]. These examples demonstrated the potential of fluorescence lifetime measurements in the analysis of artwork and art conservation research. Fluorescence lifetime measurements were found to be non-invasive and non-destructive, and, like spectrally-resolved measurements, contained more information than fluorescence intensity measurements alone.

Here, we present the development and application of a TCSPC camera-based imaging system for easel paintings. The system does not require scanning, it is portable, weighs only 4.8 kg, and is mounted on a camera tripod. It uses a pulsed visible light laser emitting at 440 nm to minimise the risk of possible damage to the painting or eyesight. A single-photon sensitive and time-resolved single-photon avalanche diode (SPAD) camera was used to perform TCSPC in parallel across its 192 × 128 pixels [24, 25]. The SPAD camera offered single-photon sensitivity across all its pixels, it was compact, easy to use, and could safely operate under ambient light. Three-exponential fluorescence lifetime analysis was performed on the data to reveal extra contrast offered by the technique. To the best of our knowledge, this is the first application of a TCSPC SPAD array and the first use of multi-exponential fluorescence lifetime imaging analysis in the field of cultural heritage study, art analysis or conservation.

## Methods

### Painting and sample preparation

For this work, a section of a painting of *The Last Supper* by an unknown artist dated to the late 18th century was used (Fig. 7). The varnish was discoloured, giving the painting a uniform brown appearance. However, the varnish removal revealed a clear vibrant blue-grey paint underneath.

Varnish removal was performed by applying industrial methylated spirit (11482874, Fisher Scientific, Loughborough, UK) as the cleaning solvent by a swab or a gel. Cotton wool (12356477, Fisher Scientific) and a wooden skewer were used to gently roll over the surface without mechanical action or with circular mechanical action. The gel was prepared by dissolving 20 ml of Carbopol C25 (CTS, Altavilla Vicentina, Italy) and 2 g ethomeen 934 (CTS) in 100 ml industrial methylated spirit and 1.5 ml deionised water. The gel was applied to the painting surface for one minute either without mechanical action or with mechanical action by agitation. Cross-section samples were taken from the surface painting using a scalpel and embedded in polyester casting resin (405–210, Tiranti, Stoke-on-Trent, UK) using a hardener (405–810, Tiranti).

The painting was imaged in areas highlighting distinctive features, mainly the edges of the varnish removal treatment areas. Images were also acquired during gradual varnish removal to monitor its progress and highlight the sensitivity of the fluorescence lifetime imaging technique to residual varnish.

### Macroscopic FLIM optical setup

**Fig. 1:**
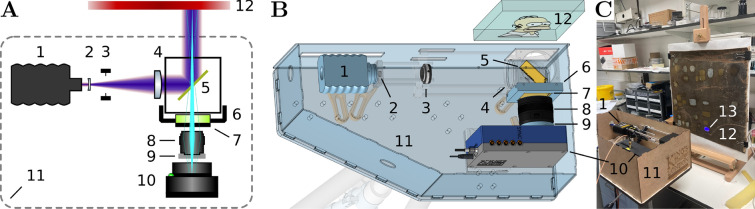
Optical setup. Panel **A** presents the diagram of the optical setup. Panel **B** shows the top-view 3D rendering of the optical setup. The critical optical components were emphasised by making the the supportive components appear semi-transparent. Panel **C** is a photo of the assembled optical setup in use with the model painting. The components in the panels are numbered: 1 − laser, 2 − concave lens, 3 − variable iris aperture, 4 − convex lens, 5 − chromatic mirror, 6 − removable emission filter holder, 7 − emission filter, 8 − objective lens, 9 − objective lens holder, 10 − SPAD camera, 11 − wooden case with its lid removed, 12 − painting, and 13 − illuminated spot on the painting. The illuminated spot was larger (≈ 35 mm in diameter) than the field of view (13.5 × 18 mm^2^) captured by the camera

The optical setup is shown in Fig. 1. It was designed in a cloud-based computer-aided design (CAD) tool Onshape (Boston, MA, USA) and the design is available in [26]. A 440 nm pulsed picosecond diode laser (DD-440 L, Horiba, Glasgow UK) was operated with a 25 MHz repetition rate, providing an excitation pulse every 40 ns. The optical pulse full width at half maximum (FWHM) was ≈ 70 ps and the average laser power ≈ 460 μW. The laser beam was expanded by a Galilean telescope consisting of a biconcave lens with focal length of –9 mm (LD2568-A, Thorlabs, Ely, UK) and a plano-convex lens with focal length of 250 mm (LA1301-A, Thorlabs). The expanded and collimated laser beam was reflected at a right angle in the horizontal plane off a 50 mm square chromatic mirror with a 480 nm high-pass cut-off wavelength (480FDL50, Knight Optical, Harrietsham, UK). The expanded laser beam illuminated the painting to excite the fluorescence. Part of this fluorescence was passed back through the chromatic mirror onto a 50 mm square 3 mm-thick GG475 Schott coloured-glass emission filter with a 475 nm high-pass cut-off wavelength (14-489, Edmund Optics, York, UK). This filter was inside a 3D-printed custom holder made by fused deposition modeling in-house. This holder enabled easy removal of the filter from the optical path for the measurement of an instrument response function (see the next section "Measurement"). The fluorescence light from the painting was collected and focused by a 24 mm focal length camera lens with f/2.8 aperture (Miranda, Japan). The camera lens holder was produced by 3D printing using laser sintering of polyamide (3D People, London, UK). The fluorescence image of the painting was projected onto the 192 × 128 pixel SPAD camera (QuantICAM, University of Edinburgh, Edinburgh, UK) [24, 25]. The SPAD camera was manufactured in a widely used 40 nm silicon complementary metal-oxide semiconductor (CMOS) process [27] and was characterised in [28]. Other applications of this and a closely related SPAD camera were described in [29–35]. Each pixel contained a single-photon sensitive SPAD and a time-to-digital converter (TDC) circuitry for measuring the photon arrival time with ≈ 38 ps precision. The useful TDC range was ≈ 35 ns. It was lower than the 40 ns pulsed laser period due to the SPAD camera pixel-dependent timing delays [28]. The photon arrival time was measured with respect to the electrical pulse synchronisation signal delivered to the SPAD camera from the laser through an RG-316 coaxial cable (Farnell, Leeds, UK). A 1 ms time window represented the exposure time, in which the camera would detect and assign a precise time-stamp to a maximum of one photon in each pixel. 100 000−250 000 repeated 1 ms exposures were measured of each sample to obtain the fluorescence decay in post-processing by histogramming the photon arrival times.

The optical system was built on a 12 mm-thick plywood base with 2 mm-thick medium density fibreboard (MDF) boards for the sides and the top, as shown in (Fig. 1) [26]. The base supported the optics while the sides and the top enclosed the system for laser safety and dust exclusion purposes. The plywood and MDF boards were custom laser-cut by (laserweb, Barnsley, UK) and polyvinyl acetate (PVA) glue (Gorilla Glue, Chorley, UK) was used to hold the case together.

The optical components were attached to the plywood base by various mechanical parts made by Thorlabs, see [26] for the details. The QuantICAM camera was mounted on a magnetic base for the ease of its removal for use with the microscope as described in section "Microscope Cross-Section Fluorescence Lifetime Imaging". The plywood base had a stainless steel 1/4”-20 thread adapter (PB2, Thorlabs) fixed below the centre of the mass of the optical system for mounting onto a camera tripod (Slik, eBay, Richmond Upon Thames, UK).

### Measurement

The painting and calibration measurements were done on samples fixed vertically in a table-top easel (06-8676, Rapid Electronics, Colchester, UK) and spaced consistently 100 mm away from the optical setup output aperture (Fig. 1**C**). The samples and the tripod with the optical setup were adjusted under bright laboratory lighting prior to the measurements. The measurements were conducted with dim ambient lighting to minimise the noise floor in the fluorescence decay data.

The calibration measurements enabled later analysis on the data from the paintings. A calibration sample was prepared by colouring a piece of office paper with a green fluorescence marker pen (70/33 Stabilo Boss, Wilko, Worksop, UK) and printing a graph paper pattern over it using a laser printer. The printer toner created a negative fluorescence contrast by concealing the underlying fluorescence marker ink. Focus was optimised by adjusting the objective lens until the sharpest graph paper pattern was formed in the SPAD camera intensity image. The dimensions of the field of view (13.5 × 18 mm^2^) was established from the number of vertical and horizontal lines spaced 1 mm apart visible in the image.

The instrument response function (IRF) is the time response of the imaging system without any fluorescence present. The IRF was used in the data analysis to correct the SPAD camera sensor timing delay, illumination beam wavefront curvature, and in the fitting of the measured fluorescence decays (Eq. (1)). The IRF was determined from the back-scattered excitation light off a white paper at a 100 mm distance, with the emission filter removed from the beam path. 10^6^ 1 ms exposures were acquired to achieve high timing accuracy of the IRF peak position required in the subsequent FLIM analysis.

Fluorescence lifetime imaging of the painting was done with the painting placed upright in the easel at a 100 mm distance. The emission filter was present in the emission beam path to reject any back-scattered light while allowing fluorescence photons to pass. Setting up the measurement and the data acquisition took between three and five minutes for each image.

### Microscope cross-section FLIM

Fluorescence lifetime imaging of the painting cross-sections was done on an inverted epi-fluorescence microscope (TE2000, Nikon Instruments, Kingston Upon Thames, UK) using the same QuantICAM sensor and data analysis as for the macroscopic surface imaging. On the excitation side was a combination of a 498 nm-edge short-pass filter (FF01-498/SP-25, Laser 2000, Huntington, UK) and a 445 nm-centered band-pass filter (ET445/30x, Cairn Research, Faversham, Kent). The chromatic mirror had a 470 nm-edge long-pass (T470lpxr, Cairn Research). The emission filter was a 550 nm-centered band-pass filter (FF01-550/88-25, Laser 2000). The excitation light was provided by a white-light supercontinuum laser (SuperK EXTREME, NKT, Brøndby, Denmark) operated with a 26 MHz pulse repetition rate. These filters and laser created similar experimental conditions to the macroscopic imaging setup, although they differed slightly in their emission filter characteristics. The microscope objective used for the imaging of the cross-sections was a 4× 0.13 NA plan-fluor objective (Nikon Instruments). For the IRF measurement [36], a 10× 0.3 NA plan-fluor objective (Nikon Instruments) was used due to its higher light-collection efficiency while the filters and laser repetition rate remained the same. The IRF measurement was acquired with a solution of fluorescein sodium (46960, Merck Life Science, Gillingham, UK) [37]. It was prepared in a saturated solution of sodium iodide (217638, Merck Life Science) in 100 mM sodium dihydrogen phosphate (10783445, Fisher Scientific, Loughborough, UK) buffer with pH 10. The freshly prepared solution was filtered through a 0.2 μm syringe filter (16534, Sartorius, Epsom, UK) to remove any precipitated fluorescein crystals. The solution was imaged inside a 8-well glass-bottom dish (80827, Thistle Scientific, Glasgow, UK). 250 000 1 ms exposures were acquired for each sample. The data processing and analysis were the same as for the macroscopic painting surface imaging and are described below (section "Data Processing").

### Microscope UV-excited fluorescence and visible light reflectance imaging of cross-sections

Cross-sections were imaged on an up-right microscope (DM4000 M, Leica Microsystems) using a 5 megapixel colour camera (DFC450, Leica Microsystems) and a dark-field contrast. Sample illumination was provided by a lamp with a mercury metal halide bulb (EL6000UV, Leica Microsystems).

### UV-excited fluorescence and visible light photography of paintings

Macroscopic photography of the painting was carried out using an 18 megapixel colour camera (EOS 600D, Canon, Uxbridge, UK) through a 50 mm focal length objective lens with f/1.8 aperture (STM, Canon) and a 390 nm−700 nm band-pass filter (Hoya UV (C), Holdan, Glossop, UK). The light source for the UV fluorescence photography were two 40 W 1198 mm-long BLB UV tubes (FL40BLB, CLE Design, London, UK) with 335 nm peak emission wavelength. Visible light photography was done under tungsten light illumination.

### Data processing

The SPAD camera raw output data was subject to processing prior to fluorescence lifetime analysis, described in depth in [28]. Briefly, the camera contained “hot-pixels” suffering from excessive dark count. The data from these pixels were not presented and were interpolated instead. The camera timing measurements were subject to pixel-dependent non-linearities and timing delays. These artefacts were corrected by a Monte Carlo method that resampled the measured photon-arrival times [28]. The resampling procedure depended on the IRF calibration measurements done on both the macroscopic and microscopic setups and a code-density map calibration measurement of the SPAD camera. The data-processing software (MATLAB and C++) and the calibration data for our SPAD array sensor is openly available online [38]. Following the processing, the data was ready for use by fluorescence lifetime analysis software TRI2 [39]. The resulting images were processed by custom MATLAB scripts (available for download in [40–45]) into the images presented in this manuscript.

### FLIM data analysis

The FLIM data analysis was done in the freely-available TRI2 software [39, 46]. A non-linear least squares Levenberg-Marquardt minimisation algorithm [47], incorporating iterative reconvolution with the IRF [48] and a Poissonian photon noise model, was used to fit the fluorescence decay data. The fit yielded the fluorescence lifetimes, amplitudes, and the background.

The data were analysed using a global three-exponential fluorescence lifetime analysis [20]. The cost function *C* for an *N*-exponential model, defined below, was being minimised.

1 $$\begin{aligned} C=\sum _{x,y}^{pixels} \left( \left( \sum _{i=1}^{N} A_{x,y,i} \times e^{-\frac{t}{\tau _i}} + Z_{x,y} \right) \circledast IRF - D_{x,y} \right) ^2 \end{aligned}$$

The cost function *C* was the sum of the squares of the residuals over all pixels [*x*, *y*] of the data set. $A_{x,y,i}$ was the amplitude of the *i*-th exponential component in pixel [*x*, *y*]. $\tau _i$ was the global fluorescence lifetime of the *i*-th exponential component representing the entire image (or a set of images) rather than a single-pixel. Thus, $\tau _i$ was the same for all pixels of the image. $Z_{x,y}$ was the background created by the ambient light and the SPAD camera dark count (noise). The model was convolved with the instrument response function $(\circledast IRF)$ before being subtracted from the measured data $D_{x,y}$ and squared. The cost function was minimised to find $A_{x,y,i}$, $\tau _i,$ and $Z_{x,y}$.

Fractional contribution images were presented in this manuscript rather than the amplitude maps $A_{x,y,i}$. Fractional contributions were not dependent on the fluorescence intensity and thus did not present the systematic patterns introduced by the nonhomogeneous laser illumination or SPAD camera artefacts. Fractional contributions $F_i$ for each fluorescence lifetime component of the *N*-exponential model were calculated from the amplitudes and the global fluorescence lifetimes as defined below.

2 $$\begin{aligned} F_i = \frac{A_i \times \tau _i}{\sum _{j=1}^N A_j\times \tau _j} \end{aligned}$$

Intensity-weighted fractional contribution images $W_i$ were created by multiplying the fractional contribution images $F_i$ with the fluorescence intensity images $I_i$.

3 $$\begin{aligned} W_i = F_i \times I_i \end{aligned}$$

The intensity-weighted fractional contribution images conveyed the information about the painting constituents through the false-colour scale and their fluorescence intensity through the brightness. They were the images presented and discussed throughout this manuscript.

**Fig. 2:**
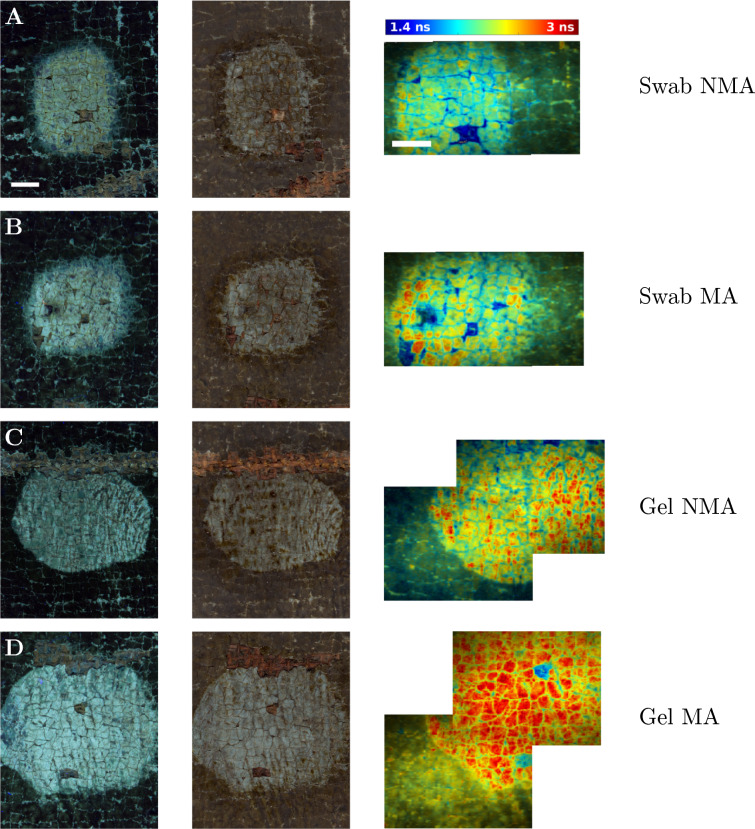
Varnish removal imaged by UV, visible light, and average fluorescence lifetime contrast. Four different varnish removal methods were used on the same painting: **A** swab with no mechanical action (Swab NMA), **B** swab with mechanical action (Swab MA), **C** gel with no mechanical action (Gel NMA), and **D** gel with mechanical action (Gel MA). In the left column are photos under UV illumination, in the middle column under white light illumination, and in the right column are the intensity-weighted average fluorescence lifetime images. The fluorescence lifetime range is 1.4 ns to 3.0 ns (false-colour scale in **A**) and the scale bars 5 mm (**A**) apply to (**A**−**D**)

**Fig. 3:**
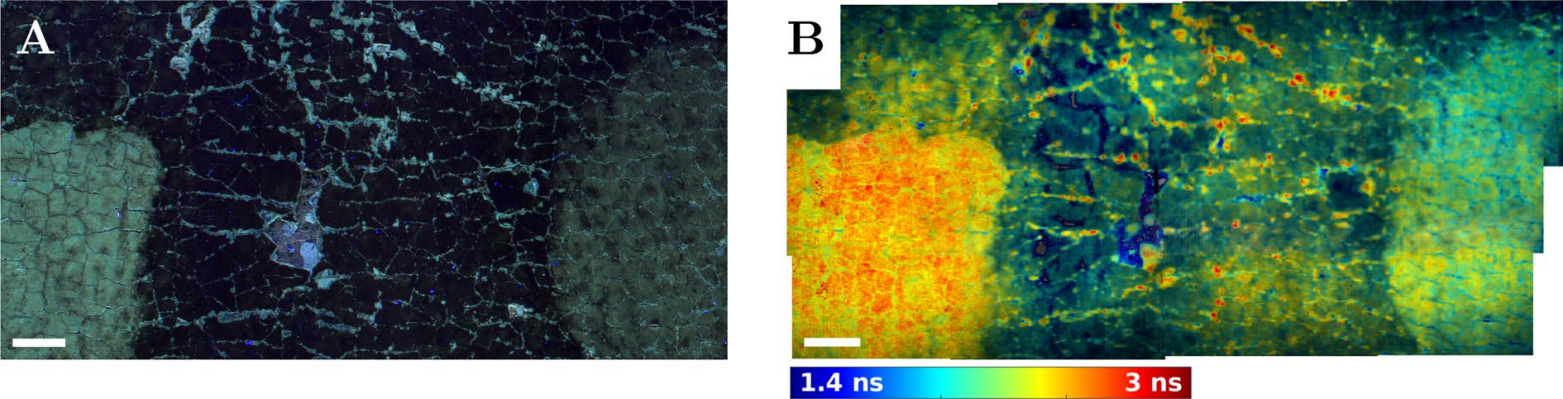
Image mosaicking. **A** Section of UV-illuminated photograph of the painting corresponding to a **B** 4 × 3 mosaic of fluorescence lifetime images. Fluorescence lifetime range is 1.4 ns to 3.0 ns. Scale bars 5 mm

The three-exponential global fluorescence lifetime fits of the measurements comparing the efficacy of four different varnish removal methods were reduced into average fluorescence lifetime images for the simplicity of their presentation in Figs. 2 and 3. The average lifetime image ${\bar{\tau }}$ was calculated from the fractional contribution images $F_i$ and their corresponding global fluorescence lifetimes $\tau _i$ [49].

4 $$\begin{aligned} {\bar{\tau }} = {\sum _{i=1}^N F_i\times \tau _i} \end{aligned}$$

## Results

The results are presented in five subsections: The developed macroscopic FLIM system and the data processing are described first. Their use in assessing the effectiveness of varnish removal by various methods is followed by the results of monitoring the gradual varnish removal. The results of microscopic examination of painting cross-sections follow next and the section concludes with the results on differentiating varnishes by their chemical composition and age.

### Design of the macroscopic FLIM system for characterisation of paintings

A 192 × 128-pixel SPAD camera with integrated pixel-level TDCs [24, 25] was used for photon detection and timing. A blue visible-light 440 nm diode pulsed picosecond laser, operating with a 25 MHz pulse repetition rate, was used as the excitation light source. The laser, SPAD camera, and optical components, i.e. lenses, filters and a chromatic beamsplitter to separate the fluorescence from the excitation light, were light-weight and compact enough to fit inside a custom wooden box (4.8 kg, 500 × 242 × 166 mm^3^) fixed on a camera tripod (Fig. 1). The system used a collimated laser beam for illumination of the imaged part of the painting. The fluorescence was separated from the back-scattered illumination light by a combination of a chromatic mirror and a long-pass emission filter. Fluorescence light collection and image projection onto the sensor of the SPAD camera was done through a 24 mm f/2.8 objective lens. The spectral sensitivity of the system ranged from 475 nm to 1000 nm with a peak at 520 nm [27].

The key and unique component of this system was the SPAD camera [24, 25]. The camera allowed wide-field TCSPC [7–9] and thus accurate FLIM without the need for moving parts to scan the excitation laser beam. The camera contained a silicon CMOS sensor with single-photon sensitivity and ≈38 ps single-photon timing accuracy in each of its 192 × 128 pixels. The camera was synchronised with the pulsed laser and measured the time between the fluorescence photon detection event and the subsequent laser pulse. This was done in all pixels independently and simultaneously over a 1 ms exposure time. 100 000–250 000 of these 1 ms exposures were acquired to collect the photon arrival times of the fluorescence photons emitted by the painting. The resulting photon arrival time histogram from each pixel represented the fluorescence intensity decay in that pixel. The data was read out and analysed after the measurement had finished. We note that it is in principle possible to obtain real-time FLIM image displays from average photon arrival times [32, 50–57], but this method was not employed here.

The excitation power required for this instrument was low, below 1 mW, in common with other wide-field single photon counting approaches. This was distributed over the field of view and avoided high local illumination intensities, as for example in raster scanning approaches. The 460 μW average excitation laser beam power distributed over the 9.2 cm^2^ illuminated area of the painting corresponded to an average irradiance of 0.05 mW·cm^−2^ at 440 nm. This was approximately 2 000 times lower than the peak solar irradiance on the surface of Earth, ≈100 mW·cm^−2^, over the whole spectrum [58].

The TCSPC method employed here could easily detect whether a fluorescence decay was mono-exponential or multi-exponential. With the 440 nm excitation wavelength none of the fluorescence decays in any of the pixels were mono-exponential in the spectral detection window used (Fig. 8). Instead, they were best fitted by a sum of three exponential decay functions (Eq. (1)) [20].

Global analysis was used in the fluorescence intensity decay fitting [20, 39, 46, 59]. The assumption was that the fluorescence lifetimes were the same in all pixels of the image and that only their fractional contributions changed due to the varying composition of the painting. The benefit of the global analysis was in the reduced uncertainty of the fluorescence parameter determination compared to fitting the same data in each pixel independently. The three-exponential global fitting yielded three representative global fluorescence lifetimes for the image (or a set of images) of the painting and their fractional contributions images. These three global fluorescence lifetimes could be associated with different materials in the painting.

The fluorescence lifetime fractional contributions images represented the spatial distribution of the painting materials (Eq. (2)). Their intensity weighting represented the fluorescence brightness distribution in the painting (Eq. (3)). The calculated global fluorescence lifetimes and their intensity-weighted fractional contributions were the results of the experiments presented throughout this work. The "Methods" section contains a more detailed description of the underlying calculations.

### A comparison of varnish removal methods

This experiment demonstrated the improved capability of FLIM to distinguish between different varnish removal methods compared to visible light or UV light fluorescence inspection. Four different methods were used to apply the solvent for varnish removal: Swab without mechanical action (Fig. 2A), swab with mechanical action (B), gel without mechanical action (C), and gel with mechanical action (D). The regions of the painting (≈ 2 cm across) at the border between the intact varnish and the treated areas were imaged. The images display a false-colour contrast of the average fluorescence lifetime ranging between 1.4 ns and 3.0 ns. The representative fluorescence decays are shown in Fig. 8. The photos of the same regions acquired under UV and visible-light illumination are also in Fig. 2.

In all four areas of the painting, the average fluorescence lifetime of 2 ns, shown in a blue-green colour, was characteristic of the aged varnish. Under UV light, the varnish appeared dark grey and under visible light brown. The varnish removal revealed the underlying paint, which had a characteristic long (red) average fluorescence lifetime, appearing light blue under UV illumination and light brown under visible light illumination. Yellow or even green rather than red colour was still present in the treatment areas after solvent application using a swab without mechanical action and to a lesser extent by a swab with mechanical action and a gel without mechanical action. These areas emphasised the presence of residual varnish fluorescence (medium lifetime, blue-green) being mixed with the underlying paint fluorescence (long lifetime, red). There was no such clear sign of residual varnish presence in the UV or visible light photography images. This demonstrated that FLIM offered a greater sensitivity and clarity when used for the detection of the presence and distribution of residual varnish. The varnish remaining in the interstices of the paint made them yellow or blue-green in the average fluorescence lifetime contrast images. These areas appeared dark-blue in the UV-illuminated and brown in the visible-light illuminated photos. The average fluorescence lifetime contrast images clearly show the ground layer as blue (short fluorescence lifetime) in the areas of paint loss.

In summary, the average fluorescence lifetime imaging of the painting surface provided a better contrast and thus clarity between the varnish, paint, and ground surfaces than UV or visible-light photography. Fluorescence lifetime images showed improved sensitivity over the other methods to residual varnish in the interstices and cracks in the paint film. The results revealed that the application of a solvent gel with mechanical action removed varnish more effectively than the other three application methods used on the painting.

This experiment also demonstrated the potential for mosaicking several fluorescence lifetime images to cover a larger field of view. The mosaicking was done by adjusting the camera tripod height and/or shifting the easel with the painting in a horizontal direction.

A mosaic of 4 × 3 fluorescence lifetime images was acquired to demonstrate the feasibility of imaging larger areas of the painting surface without sacrificing the image resolution (Fig. 3). It was compared to UV-light photography. The images revealed the different effectiveness of varnish removal in the two treatment areas. The lower-left treatment area was homogeneously red, a hallmark of efficient varnish removal, unlike in the right treatment area, which appeared green to yellow. The red spots and the cracks, indicative of varnish fracturing, and the areas of blue ground or uncovered paint correlate with the bright areas in the UV-fluorescence photograph. The UV-photography could not unambiguously distinguish between the areas of paint loss and varnish loss. The ground appeared in the same colour as the paint in the UV-fluorescence image while they were yellow and deep-blue, respectively, in the fluorescence lifetime image. This again demonstrated the advantage of using average fluorescence lifetime imaging over UV-illumination photography for the differentiation of the varnish, paint, and ground materials.

### Gradual varnish removal

**Fig. 4:**
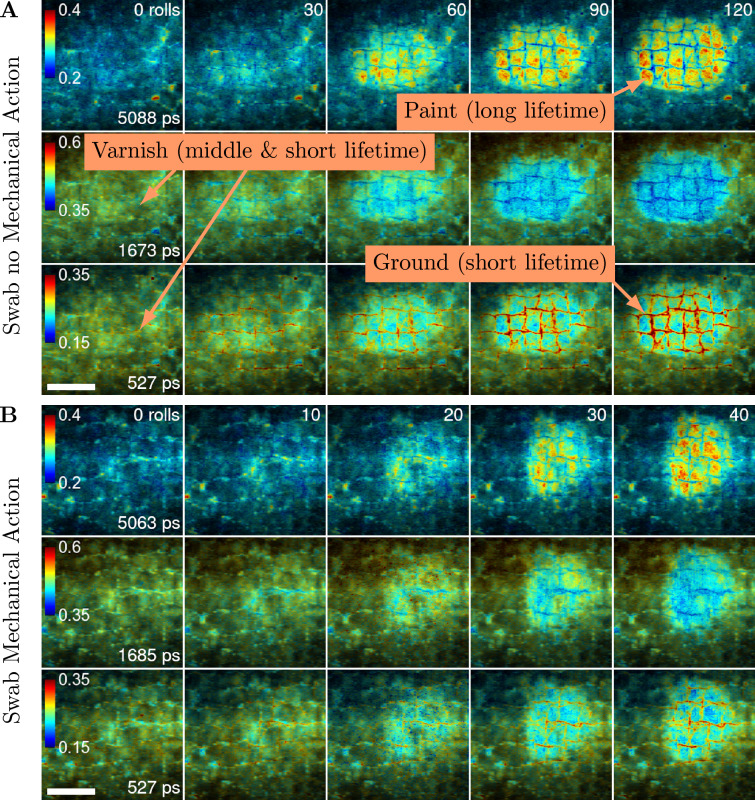
FLIM of gradual varnish removal. Rows of images showing the intensity-weighted fractional contributions as they developed during the gradual varnish removal treatment. The representative fluorescence lifetimes and the false-colour scale representing the fractional contributions for each row are shown in the images in the first column. The numbers of solvent applications are indicated in the top-right corners in the first rows of the images in **A** and **B**. **A** The solvent was applied to the painting by a swab without mechanical action. In total, 120 rolls of the swab were performed with 30 rolls between imaging. **B** The solvent was applied by a swab with mechanical action in a total of 40 rolls with 10 rolls between imaging. Text boxes and arrows in **A** highlighted the materials of the painting assigned to their characteristic lifetimes. Scale bars 5 mm

To visualise the varnish removal process in more detail, solvent was gradually applied by a swab and the FLIM images were recorded between the successive applications. The solvent was applied to the painting using a swab without mechanical action in a total of 120 rolls and 30 rolls between imaging (Fig. 4A). To compare different application methods, it was applied to a separate area of the painting by a swab with mechanical action in a total of 40 rolls and 10 rolls between imaging (Fig. 4B). Three-exponential global fitting was applied to the fluorescence decay data to separate three fluorescence lifetimes representative of the painting. The solvent application without mechanical action yielded global fluorescence lifetimes of 5088 ps, 1673 ps, and 527 ps. Using mechanical action, comparable global fluorescence lifetimes of 5063 ps, 1685 ps, and 527 ps were obtained. The intensity-weighed fractional contribution images (Eq. (3)) for the three fluorescence lifetime components are presented in Fig. 4 encoded in a false-colour scale: The blue colour indicated a low contribution of about 20% for the long lifetime, 35% for the medium lifetime and 15% for the short lifetime. This changed through green and yellow to red, which highlighted the areas with high contributions (40% for the long lifetime, 60% for the medium lifetime and 35% for the short lifetime) of the given global fluorescence lifetime components.

The three-exponential fits of the fluorescence decays enabled the identification and separation of the main components of the painting (Fig. 4). The longest fluorescence lifetime components (5088 ps or 5063 ps) started appearing as the varnish removal progressed and revealed the underlying paint. This is indicated by the increased area of the red and yellow colour in the FLIM images in the top row of Fig. 4A and B. The short (527 ps) and more so the medium (1673 ps or 1685 ps) fluorescence lifetime components could be attributed to the varnish. Their fractional contributions were decreasing during the varnish removal treatment, as indicated by the increase of blue colour in the FLIM images of the short and medium lifetime components in the middle and bottom rows of Fig. 4A and B. The short (527 ps) fluorescence lifetime component was mainly attributed to the ground layer, whose fluorescence increased in the cracks as the varnish was being removed.

FLIM of the gradual varnish removal showed this process in an unprecedented detail and clarity. Moreover, the application of TCSPC enabled three-exponential analysis of the measured fluorescence decays. It resulted in three images for one measurement, each showing the fractional contributions of one of the fluorescence decay components. The successive applications of the solvent caused changes to the fractional contributions of the three fluorescence lifetimes as the varnish was being removed. The three images for each sample enabled a more accurate interpretation and identification of the painting materials, including the varnish, when compared to the average fluorescence lifetime presentation (Fig. 2).

### Microscopic FLIM of painting cross-sections

**Fig. 5:**
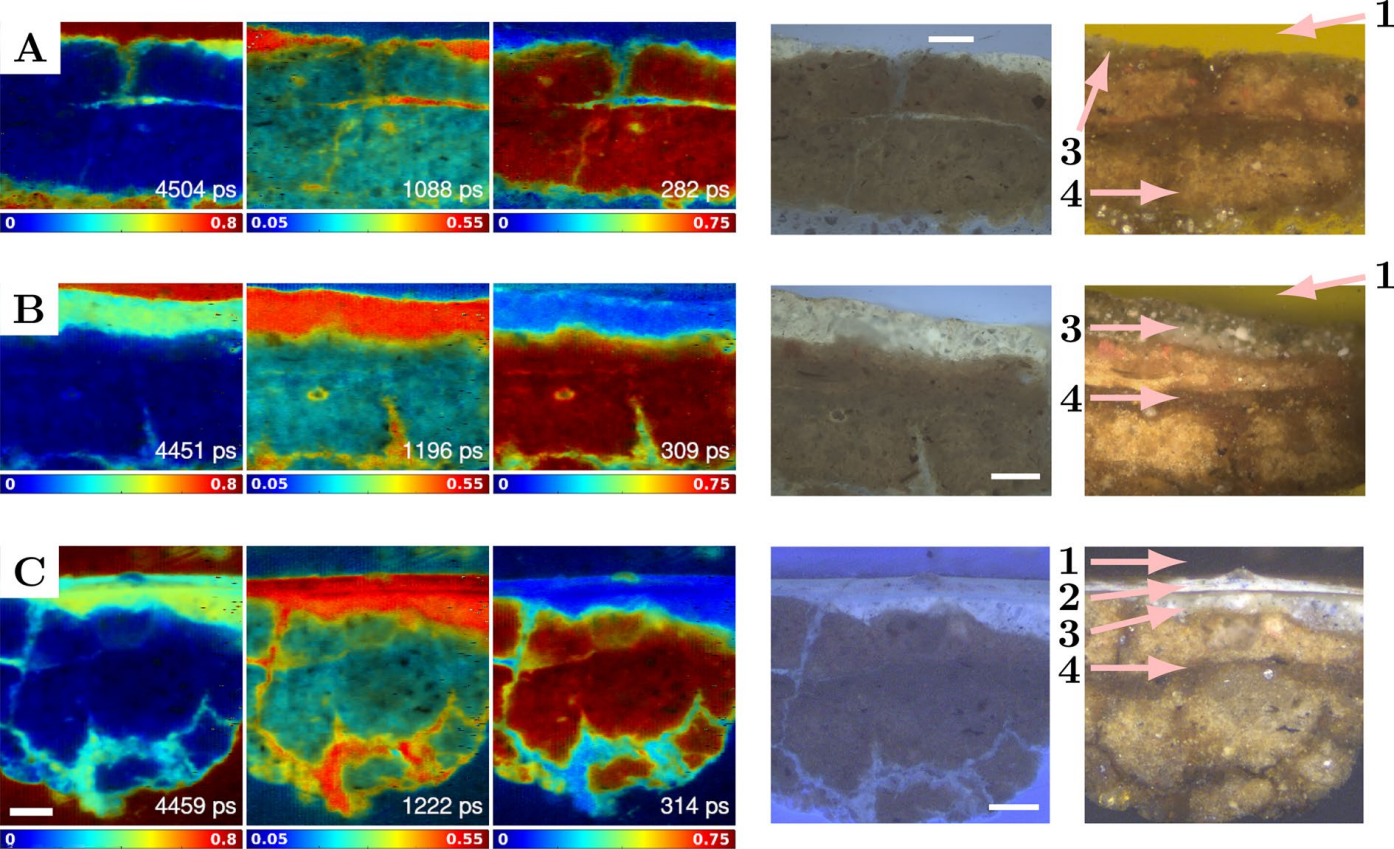
Painting cross-sections. Samples from the painting were extracted where solvent was **A** applied by a swab with no mechanical action, **B** applied by a swab with mechanical action, and **C** not applied to leave the varnish intact. The left three columns show the intensity-weighted fractional contributions. The representative global three-exponential fluorescence lifetimes are shown in the bottom right corner of each inset. The ranges of the fractional contributions and the false-colour scales are underneath each inset. The fourth column shows the UV-excited broadband visible fluorescence images of the cross-sections. The rightmost column shows dark-field reflectance images with the labels and arrows pointing to the different painting layers. 1 is the embedding resin, 2 is the varnish, 3 is the paint, and 4 is the ground. Scale bars 100 μm

A widely used approach to investigate painting stratigraphy is by taking microsamples through the layers and embedding them in resin that is ground at right angle to the surface. The revealed layers in the cross-sections were examined by FLIM in this study. Samples were taken from the areas displayed in Fig. 2A and B and embedded in a polyester resin.﻿ The cross-sections were imaged using a wide-field fluorescence lifetime microscope equipped with the same SPAD camera used for the macroscopic surface FLIM. The resulting FLIM images were compared to the microscopy images obtained in reflectance dark-field white-light and UV-light illumination modes. The three cross-sections exhibited similar fluorescence lifetime properties, and, as in section "Gradual Varnish Removal", the fluorescence decays were fitted using a global three-exponential model and presented as images of intensity-weighted fractional contributions in Fig. 5. The cross-sections in Fig. 5A and B had the varnish removed while the cross-section in Fig. 5C featured an intact layer of varnish.

A long fluorescence lifetime of ≈ 4500 ps, a medium lifetime of ≈ 1200 ps, and a short lifetime of ≈ 300 ps were found. The long lifetime (first column, red) was principally assigned to the embedding resin because the fluorescence emanated from a region where no painting sample was present. The resin formed the upper-most layer in all cross-section images in Fig. 5. The paint and varnish layers (Fig. 5C) found beneath the resin layer were most pronounced in the middle lifetime component (second column, red). Their contribution to the long lifetime component (first column, yellow/green) was also elevated. Interestingly, when embedded in the resin, the varnish and the paint were barely discernible by their fluorescence lifetime, unlike in the macroscopic surface imaging (Figs. 2 to 4). The lowest layer, consisting of the ground, was contributing the most by its short lifetime component (third column, deep red). This was consistent with the results of the macroscopic surface imaging. Conventional UV-fluorescence microscopy and dark-field reflectance microscopy with visible light were also performed on the same cross-sections. Consistent with the fluorescence lifetime imaging, the UV-fluorescence of the varnish layer appeared very similar to the underlying paint (Fig. 5C), once embedded in the resin.

A quantitative comparison between the results of the microscopic cross-sections FLIM and the macroscopic surface FLIM was hindered by the additional strong fluorescence emitted by the embedding resin in the cross-sections samples. Consequently, the three global fluorescence lifetimes differed between the two imaging modalities and the contrast between the paint and the varnish was lost in the images of the cross-sections (Fig. 5C). Nevertheless, qualitatively the results of the microscopic and macroscopic analyses showed similar features.

### Ageing effects on fluorescence lifetime of natural resin varnishes

The results presented above were obtained from a historical painting of unknown composition and physical history. They were complemented by the results presented below of a FLIM study on the samples of known varnishes and ageing histories. The varnish samples were produced and lent by Tate (London, UK). They were composed of titanium white paint coated with layers of either dammar or mastic natural resin varnishes. The samples were aged in two ways. Those undergoing ambient ageing were kept in ambient indoor daylight since their creation in 1988 until 2013 when they were transferred into dark storage. The samples undergoing accelerated ageing were additionally exposed to daily sunlight and up to 30 °C temperature in a weatherproof light-box over a three-month summer period. The cumulative light dose was estimated to be equivalent to 61 years of museum light exposure by reciprocity [60].

**Fig. 6:**
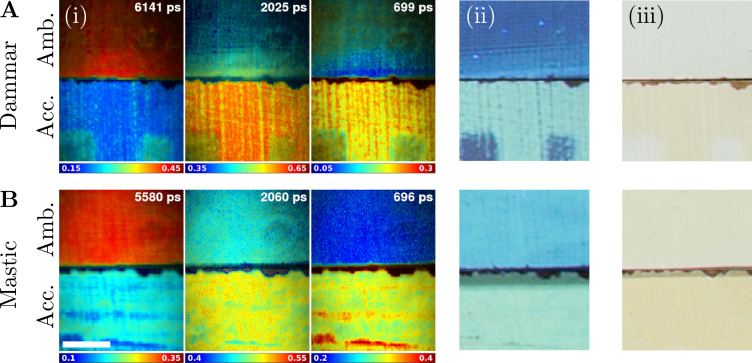
Natural resin varnish samples. **A** dammar and **B** mastic resin-based varnish samples following ambient ageing (Amb., top row) and accelerated ageing (Acc., bottom row) are displayed. Each row contains images of (i) intensity-weighted fluorescence lifetime fractional contributions, (ii) UV-excited broadband fluorescence intensity, and (iii) visible-light illumination photographs. The ranges of the fractional contributions and the false-colour scales are below each fractional contribution image. The characteristic global fluorescence lifetimes are in the top-right corners. **A** The visible rectangles are the consequence of historical varnish removal treatment. (i) Elliptical rings visible in the top-right quadrant result from the inhomogeneous illumination, which influences only the grey-scale intensity weighting and not the colour-scale fluorescence lifetime fractional contributions. (i, ii) Visible streaks are caused by variable varnish layer thickness due to its application by brush. Scale bar 5 mm

The varnish samples were imaged by FLIM and the data were analysed using three-exponential global analysis. Fluorescence lifetime fractional contribution and fluorescence intensity changes were compared between the varnish samples undergoing ambient or accelerated ageing (Fig. 6). Additionally, phasor analysis was applied to the same fluorescence intensity decay data to demonstrate the feasibility of this simple non-fitting data analysis method to discriminate the natural resin varnishes by their age (section C, Fig. 9). The varnish samples were also photographed under visible and UV-light.

Dammar varnish (Fig. 6A) fluorescence intensity was increased in the varnish sample subject to accelerated ageing compared to the sample aged in ambient conditions. This is evident from the intensity-weighted fluorescence lifetime fractional contribution images (i) and UV-light illuminated photography (ii). This change in the fluorescence intensity was concurrent with a considerable drop in the fractional contribution of the long lifetime component (6141 ps) and rise in the short lifetime component (699 ps) and the medium lifetime component (2025 ps) as shown in Fig. 11A. The varnish undergoing accelerated ageing was more yellowed, as evident in (iii), consistent with the observed behaviour of painting varnishes.

Mastic varnish (Fig. 6B) exhibited similar behaviour to dammar. The fluorescence intensity from the mastic experiencing accelerated ageing was higher than from mastic aged in ambient conditions, which in turn was higher than from dammar aged in ambient conditions. However, the difference in the fluorescence intensities of the two mastic samples was not as pronounced as with dammar. The changes to the fluorescence lifetime of mastic were similar to dammar. The fractional contribution of the long lifetime component (5580 ps) dropped considerably as a result of the accelerated ageing. The middle lifetime component (2060 ps) increased slightly, while the short lifetime component (696 ps) increased considerably (Fig. 11B).

The samples subject to accelerated ageing displayed stripes in the fractional contribution images not apparent in the images of the samples subject to ambient ageing. These were likely the consequence of variable varnish thickness due to its application by brush. Areas of thicker varnish offered protection from the sunlight to the deeper layers of the varnish, which in turn aged more slowly. Notably, the characteristic global fluorescence lifetime components were within 10 % of each other for the mastic (5580 ps, 2060 ps, and 696 ps) and dammar (6141 ps, 2025 ps, and 699 ps) varnishes. The visible light photography revealed the dammar and mastic varnishes following accelerated ageing were yellower than those aged in ambient conditions.

## Discussion

### The instrument

A compact and portable system for multi-exponential fluorescence lifetime investigation of paintings was designed and built for the purpose of monitoring varnish removal treatment on easel paintings. Below, the potential improvements, modifications, and their impact are discussed.

A visible light laser was chosen for the safety of the painting materials and the users. However, a UV pulsed picosecond laser or multiple excitation lasers could provide additional information about the painting materials [61]. Laser replacement would require a redesign of the illumination optics. The use of a larger broadband or tunable laser, like a supercontinuum source, instead of the diode laser would compromise the portability the system. The illumination beam was created by expanding the laser output with a Galilean telescope. The resulting illumination was non-homogeneous as the beam had an elliptical shape with a near-Gaussian profile. A custom-made diffractive optical element instead of the concave lens in the telescope could be designed to produce a homogeneous sample illumination (top-hat profile).

The optical system was built on a 12 mm-thick plywood breadboard with 2 mm-thick MDF sides to minimise the cost and weight. The unorthodox choice of wood to build the optical setup proved successful, although the bridging mounting clamps used to attach the optical components caused warping of the plywood and optical alignment difficulties. Using non-bridging (flat bottom) mounting mechanisms would alleviate the problem. Alternatively, a rigid aluminium breadboard could be used instead, adding additional cost and weight. The objective lens holder was produced as a separate part by 3D printing. Having the objective lens separated from the camera made the assembly insufficiently rigid for easy focusing of the image. A 3D-printed SPAD camera case incorporating the objective lens holder would overcome this issue.

The discussed improvements and modifications could widen the utility and improve the ease of use of the instrument. However, the presented minimal setup proved successful in demonstrating the advantages of using a SPAD camera for painting investigation compared to earlier approaches [4, 11–16, 18, 19, 21–23]. This included the combination of macroscopic imaging, small footprint, portability, use of relatively safer visible-light low pulse-energy laser for excitation, application of time-correlated single-photon counting for accurate multi-exponential analysis, robust and compact solid-state light detector, and potentially low manufacturing cost.

The macroscopic surface FLIM and the microscopic cross-section FLIM used the same SPAD camera. The camera was mounted on a magnetic kinematic base, allowing an easy transfer between the two systems without any need for optical realignment. This demonstrated the wider applicability of the SPAD camera and could enable the development of microscopic and macroscopic imaging systems for arts study and conservation based on the same light detector.

Dedicated SPAD cameras can be effectively used for time-gated FLIM [62, 63]. Time-gated SPAD cameras benefit from their simpler architecture and thus can be made with a higher number of pixels than TCSPC SPAD cameras, such as the one used in this work. They could enable imaging systems with improved spatial image resolution and/or larger field of view at the expense of reduced photon efficiency, measurement accuracy, and longer acquisition time. This could make accurate analysis of short lifetimes or multi-exponential fluorescence decays more challenging, if not impractical. Time-gated SPAD cameras offer distinct advantages over time-gated image-intensified cameras, previously used in the FLIM of cultural heritage [4, 12–16, 18, 19]. They are less complex, more compact, offer improved timing control and jitter performance, and are not susceptible to damage by overexposure to light. Thus, they could enable systems for time-gated FLIM of cultural heritage with improved characteristics.

Time-of-flight SPAD sensors, like the one used in this study, are commonly used in light detection and ranging (lidar) to reconstruct a 3D scene [29, 34, 64]. In cultural heritage studies, lidar was used for 3D fluorescence studies of architectural reliefs [65] or ancient excavations [66]. The data processing done in the presented work used an algorithm (section "Data Processing"), which corrected for timing delay from an IRF measurement [28]. The same algorithm could be used for simultaneous fluorescence lifetime analysis and 3D object surface shape reconstruction by lidar. Therefore artworks, such as sculptures or architectural reliefs, could be reconstructed with their fluorescence lifetime signature superimposed on their 3D relief model. This could be used for remote non-invasive study of different artworks composition or early detection of biodegradation [66].

### The method

In this study, an area of 13.5 × 18 mm^2^ was imaged that offered the opportunity to evaluate the efficacy of different varnish removal treatments on a scale typical in conservation diagnostics. The measurement area could be increased by changing the optical magnification of the laser beam expander (Galilean telescope), positioning the painting further away from the system, and appropriately refocusing the objective lens. However, a larger field of view might require a more powerful laser source and would lead to the loss of spatial resolution and thus the ability of the system to detect detailed structures in the paintings. A more appropriate way to increase the field of view would be through image mosaicking, as shown in Fig. 3. This could be done by using a motorised painting easel and automating its movement in synchrony with the data acquisition.

This study advanced the field of painting surface and cross-section imaging by fluorescence lifetime through the use of multi-exponential analysis of the fluorescence intensity decays. Global fitting of the data [20, 39] provided a framework for a new way of fluorescence lifetime data interpretation compared to the single-exponential fitting or non-fitting methods used in cultural heritage study in the past [4, 11–16, 18, 19, 21–23]. Global fitting reduced the experimental measurement time by requiring less measurement data. It worked with a smaller number of degrees of freedom compared to the conventional TCSPC fluorescence lifetime analysis and resulted in high quality images even with a moderate number of photons per pixel (10 000−100 000). The measured data was separated into three representative fluorescence lifetimes and their fractional contribution images. Each fractional contribution image could be interpreted as a representative map of distribution of distinctive painting materials to simplify and strengthen the interpretation. The three images resulting from the multi-exponential analysis could be reduced to a single image by calculating the average fluorescence lifetime (Eq. (4), Figs. 2 and 3). This simplification could be useful to the conservator when the contrast in the average fluorescence lifetime image would be sufficient to discriminate the different painting materials.

### Varnish removal

The FLIM of four different varnish removal treatment areas shown in Fig. 2 has demonstrated the improved contrast and sensitivity to residual varnish compared to the UV and visible light photography. The gradual varnish removal treatment progress in Fig. 4 was visualised with great clarity and detail by FLIM, which could help conservators optimise the method. A disagreement in the signature fluorescence lifetime was found between the surface macroscopic images and the microscopic images of the cross-sections. This could be explained by the additional contribution of the embedding resin to the overall fluorescence of the sample. Consequently, the varnish and the underlying paint became difficult to discern in the cross-section FLIM microscopy, although they exhibited a high contrast in the surface macroscopic FLIM. Improvements might include the exclusion of the resin from the fluorescence lifetime analysis by processing a region of interest containing the painting sample but not the resin. However, the embedding resin fluorescence would still contaminate the painting sample fluorescence inside the region of interest due to the considerable out-of-focus blur from the thick cross-section samples. Cross-section FLIM on a confocal TCSPC microscope could be more suitable thanks to its optical sectioning capability. It would reject all out-of-focus light, generate crisper and sharper images and thus allow the analysis of the painting cross-sections without the contaminating resin fluorescence.

The varnish removal treatments revealed that fluorescent materials with distinctive fluorescence lifetimes were present in the painting (Figs. 2 to 4). The data from the dammar and mastic varnishes implied different fluorescence lifetime signatures dependent on their ageing conditions (Fig. 6). A systematic study on a wider range of varnishes and other painting materials could enable non-invasive material identification and characterisation of paintings by FLIM [4]. Using spectrally-resolved FLIM [61] or combining FLIM with other analytical techniques, such as mass spectroscopy, Fourier transform infrared spectroscopy or Raman spectroscopy [21] could improve the accuracy of assigning the representative fluorescence lifetimes to specific painting materials.

## Conclusion

This work demonstrated that fluorescence lifetime imaging of varnish could provide better contrast, sensitivity, and specificity compared to visible light or UV-excited fluorescence photography.

Each measurement took only 2–5 minutes including the setup. The images captured a field of view compatible with the size of a typical varnish removal treatment area and had a resolution sufficient to resolve sub-millimeter features on the painting surface, such as paint interstices. The field of view could be extended by mosaicking to capture larger areas of the painting. The data analysis could produce average fluorescence lifetime images for the ease of their interpretation or multi-exponential global fluorescence fractional contribution images to separate various painting materials. The system demonstrated improved sensitivity and contrast to residual varnish during varnish removal treatment compared to UV-excited fluorescence photography. It was able to distinguish varnishes from other painting materials and varnishes of different age and compositions.

This work achieved several technical breakthroughs in the application of FLIM for the study of art. To the best of our knowledge, this is the first example of wide-field macroscopic and microscopic FLIM of cultural heritage using SPAD arrays. Moreover, this work used a three-exponential fluorescence intensity decay analysis, which provided a more accurate model for the complex decays of the fluorescence from the paintings: it retrieved extra detail and contrast by presenting three complementary images, rather than blending the data into a single image, as done previously. Furthermore, the instrument was smaller, safer, portable, and more robust than the previously reported alternatives for fluorescence lifetime analysis. Future developments should involve automation for large area scanning, application to more varied pieces of art, and a quantitative comparison to multi-spectral or hyper-spectral imaging.

## Supplementary information

The supplementary data for this manuscript include a photograph of the painting used in this study, representative fluorescence intensity decays from the measured samples, phasor analysis of the data, and histograms of fractional contributions.

## Declarations

**Fig. 8:**
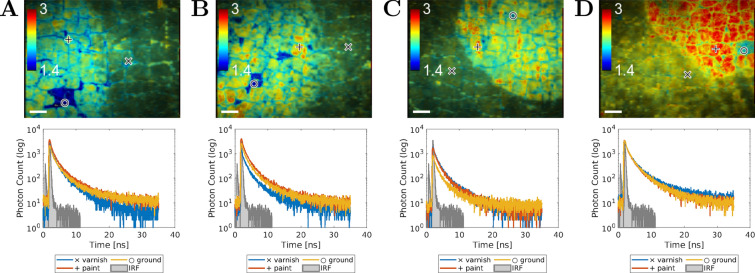
Representative fluorescence decays of varnish removal FLIM. The average fluorescence lifetime contrast images, same as in Fig. 2, are in the top row. Three example pixels representing varnish (blue line), paint (red line), and ground (yellow line) were marked by a cross ($\times$), a plus ($+$), and a circle ($\circ$), respectively. The fluorescence decays in these pixels are plotted in the graphs in the lower row. The IRF is shown in grey. The columns of the figure contain data taken after the following solvent application methods: **A** swab rolling with no mechanical action, **B** swab rolling with mechanical action, **C** gel application with no mechanical action, and **D** gel application with mechanical action. The fluorescence lifetime ranges and false-colour scales are in the top-left corner of each panel. Scale bars 2 mm

**Fig. 9:**
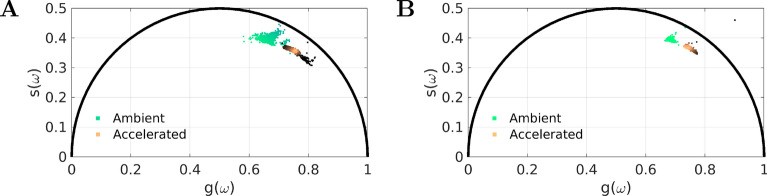
Phasor analysis separated varnish samples by their ageing conditions. Point clouds from phasor analysis of fluorescence intensity decays were produced for samples of **A** dammar and **B** mastic natural resin varnishes. Varnish aged under ambient conditions is shown in green and varnish undergoing accelerated ageing in brown. Points closer to the right end of the unity semicircle (accelerated ageing) correspond to shorter lifetimes. Points inside the unity circle (all) indicated multi-exponential fluorescence intensity decays. Plots were derived from the same data used in producing Fig. 6. g(ω) is the real part and s(ω) is the imaginary part of the Fourier transform of the fluorescence intensity decay in each pixel

**Fig. 10:**
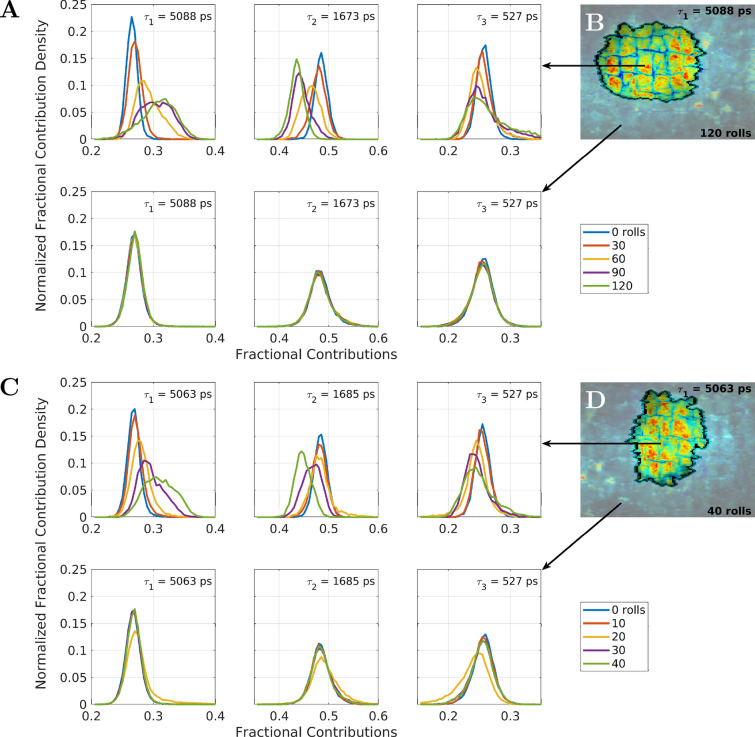
Fractional contribution histograms of gradual varnish removal. **A** Fractional contribution histograms were created from the image data acquired during the gradual varnish removal done by solvent application using a swab without mechanical action. The top row shows three sets of histograms for each representative lifetime from the inside of the area of the image highlighted by the black line in **B**. The bottom row of histograms is from the outer control area, where no solvent was being applied. **C, D** Same as above, but done on the image data acquired during solvent application using a swab with mechanical action. All histograms were normalised to make their integrals equal to 1

**Fig. 11:**
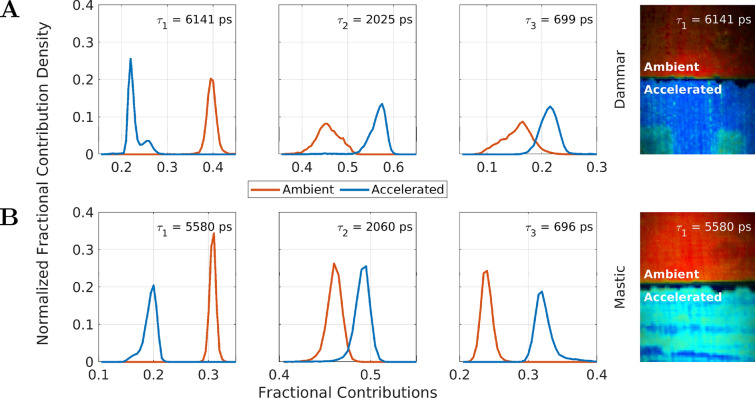
Fractional contribution histograms separated varnish samples by their ageing conditions. **A** Fractional contribution histograms were created from the FLIM data of dammar varnish subject to ambient (red lines) or accelerated (blue lines) ageing conditions. The graph panels show the fractional contribution histograms for the three different representative lifetimes (top-right corner). The image on the right shows the fractional contribution contrast for the longest lifetime component. **B** The same as above, but based on the mastic varnish FLIM data. All histograms were normalised to make their integrals equal to 1

- The data analysis code:
  *: Project name: SPAD Linearization Code.
  *: Project home page: https://github.com/jnedbal/SPADcorrection.
  *: Archived version (includes calibration data): https://doi.org/10.18742/20411565 [38].
  *: Operating systems: Linux 64-bit, Windows 64-bit, MAC OS X (untested).
  *: Programming language: MATLAB, C++.
  *: Other requirements: *DIPimage* library (https://diplib.org/), Slimcurve library MATLAB wrapper (https://github.com/jnedbal/slimcurve).
  *: License: BSD 2-Clause, GNU GPL 3 for Slimcurve.
  *: Any restrictions to use by non-academics: See *DIPimage* and Slimcurve licenses for the details.
- The data and data processing scripts used to create Figs. 2 and 8 are available in [41].
- The data and data processing scripts used to create the image mosaic in Fig. 3 are available in [42].
- The gradual varnish removal data and data processing scripts used to create Figs. 4 and 10 are available in [43].
- The cross-section data and data analysis scripts used to create Fig. 5 are available in [44].
- The data acquired with resin varnish samples aged in ambient and accelerated conditions and the data analysis scripts used to create Figs. 6, 11 and 9 are available in [45].
- The instrument response function calibration measurement data for the macroscopic surface imaging are available in [67] and for the microscopic cross-sections imaging in [36].

The 3D CAD model of the optical setup, designed in Onshape, is available for viewing and editing in [26].

## Figures and Tables

**Fig. 7:**
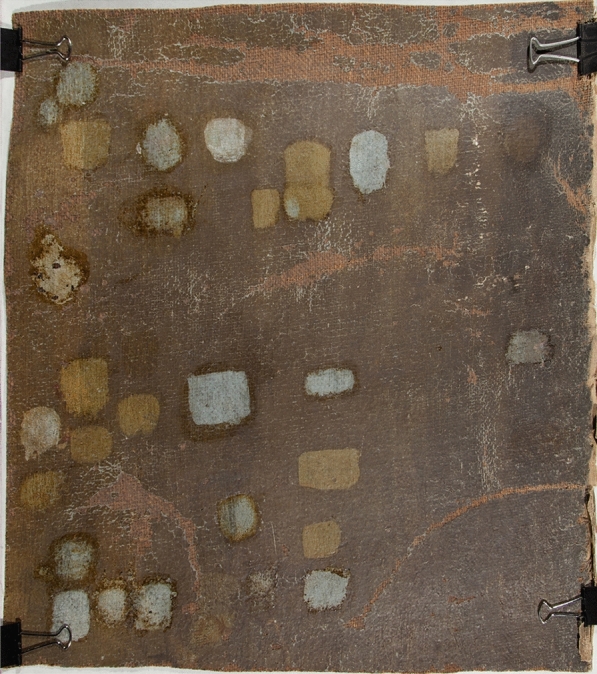
Photograph of the studied painting. The painting is dotted with varnish removal treatment areas, some of which are historical and others result of the work presented here

## Data Availability

The datasets supporting the conclusions of this article are available in the figshare repository https://doi.org/10.18742/c.6137130 [40].

## Appendix A painting photograph

A part of a canvas from a painting of The Last Supper by an unknown artist, dated to the late 18th century, was used in this study. A photograph of this painting is shown in Fig. 7.

## Appendix B fluorescence decays

The fluorescence decays from the varnish removal FLIM (Fig. 2) are displayed in Fig. 8. The figure shows that the decays were not mono-exponential. Moreover, they had sufficient photon counts for multi-exponential fluorescence decay fitting, with peak amplitudes in excess of 1 000 counts. The instrument response function was well resolved. It included an earlier secondary peak caused by light scattering from the chromatic mirror placed into the beam path. This secondary peak was disregarded in the fitting. Noticeable was the different background from the varying dark counts of the different pixels. This is not found in laser-scanning TCSPC microscopes, which use the same detector and integration time for each pixel. It is however typical of the SPAD cameras with multiple pixels.

## Appendix C phasor analysis

Fluorescence intensity decay fitting is not the only technique available for fluorescence lifetime analysis and data presentation. Phasor analysis reduces the complex decay into two points using a Fourier transform [68]. These points are plotted for each pixel of the image to create a density cloud plot instead of an image with a fluorescence lifetime contrast. The phasor analysis process is explained in detail in a technical note [69] and a protocol [70]. Its recent application to art material analysis can be found in [21, 22]. The advantages of the phasor analysis are in its rapid computation speed compared to the fitting methods, no need for the selection of an implicit decay model and no requirement for an instrument response function. However, it reduces the measurement data such that the spatial information is lost. This makes phasor analysis more suitable for automated or real-time data processing or when applied to spatially homogeneous samples.

The phasor plot analysis was suitable for the varnish samples aged under the accelerated and ambient conditions (section "Measurement" Fig. 6) as they featured only little spatial contrast. The same fluorescence decay data used in producing Fig. 6 was subject to the phasor analysis (Fig. 9). The data were divided into parts depending on their ageing conditions and a separate phasor analysis was applied to each part. The results were overlaid into a single graph for each varnish sample. The different ageing conditions led to a clear separation in the resulting phasor plots. The point clouds for the sample subject to accelerated ageing (brown points) were closer to the right end of the unity semicircle, indicating shorter average lifetime compared to the ambient-aged controls (green points). This was consistent with the results of the three-exponential global analysis, which found a rise in the short lifetime component and a drop in the long lifetime component as a result of the accelerated ageing. All point clouds were confined inside the unity circle. This indicated that the decays were multi-exponential, which was in agreement with the global three-exponential analysis, too. The point clouds for the dammar varnish were more widely spread around their centre of gravity compared to the mastic. This could have been due to the lower fluorescence yield of dammar compared to mastic and the additional heterogeneity introduced by the presence of the varnish removal treatment areas in the dammar sample. Consistent with the three-exponential fluorescence lifetime analysis, the phasor plot was capable of separating the varnishes by the ageing conditions but was unable to discriminate between the two resins, as their fluorescence intensity decays under the same ageing conditions were similar.

The phasor analysis approach could offer a quick insight into the material properties in homogeneous parts of a painting and discriminate between different kinds of materials with the caveat that spatial information was lost. It may be suitable for rapid fluorescence lifetime estimation for real-time display or for automated data analysis or image segmentation with examples in [21, 22]. However, the loss of spatial fluorescence lifetime contrast and the inability to separate multiple fluorescence lifetime components limited the usefulness of phasor analysis for the analysis of complex painting surfaces.

## Appendix D fractional contributions histograms

Histograms of fractional contributions offered an alternative approach to displaying the trends in the three-exponential fluorescence lifetime data. These histograms represented the proportions of pixels in an image that showed similar fractional contributions. The histograms reduced the complex image data into simple graphs that helped visualise the trends and the relative sizes of the features in the images. The histogram presentation was implemented on the gradual varnish removal data (section "Gradual Varnish Removal" Fig. 4) and on the comparison of varnish ageing conditions (section "Ageing Effects on Fluorescence Lifetime of Natural Resin Varnishes" Fig. 6). In both examples, the images were segmented to allow comparison between their different parts.

Gradual varnish removal was conducted by swab application of solvent without mechanical action (Fig. 10A, B) and with mechanical action (Fig. 10C, D). The automatic image segmentation on the gradual varnish removal data was based on the images acquired after the final varnish removal treatment (Fig. 10B, D). The areas of the removed varnish were outlined by black lines with the varnish-free area inside and the intact varnish outside. Identical segmentation was applied to all five images of each sample acquired during the gradual varnish removal treatment. The corresponding histograms are presented in Fig. 10A, C. The histograms of pixels within the varnish removal treatment area are in the top rows and the control histograms of pixels outside the treatment area are below. In the control area, the histograms show that the fractional contribution distribution was symmetrical and unchanged prior, during, and after varnish removal for all three representative fluorescence lifetimes and both varnish removal processes. Meanwhile, the histogram shapes evolved inside the treatment area throughout the varnish removal process. For the longest fluorescence lifetimes (first column), the varnish removal led to histogram broadening and shift to the right, indicative of the increase in the longer lifetime component, but also heterogeneity in the image contrast. The medium fluorescence lifetime histograms (middle column) shows a gradual shift to the left, without any change to the peak width. This was consistent with a homogeneous decrease in the contribution of this component as the varnish was being removed. The shortest lifetime component histograms (right column) shows a bimodal behaviour, where the main peak was shifting to the left and a shoulder was forming to the right. The peak shift to the left is indicative of the gradual loss of this component as a result of the varnish removal treatment. The shoulder formation, especially pronounced in Fig. 10A, implies a heterogeneous image feature (ground) being revealed during the varnish removal treatment. The histogram analysis interpretation was consistent with the interpretation of the image data in section "Gradual Varnish Removal" and Fig. 4.

Similar histogram analysis was applied to the natural varnish samples subject to ambient or accelerated ageing. The histograms presented in Fig. 11 were calculated for all three representative fluorescence lifetime components, the two varnish samples, and the two ageing conditions. The histogram peak positions were well distinguished between the two varnishes and their ageing histories. The dammar sample after accelerated ageing, especially pronounced in the long lifetime component, had a bimodal distribution (Fig. 11A, left panel). This was due to the presence of thinned areas of varnish with different fluorescence decay properties, created during earlier varnish removal treatment. A less pronounced bimodal distribution was also visible in the mastic sample after accelerated ageing (Fig. 11B), where brush stroke features lead to a contrast in the fractional contribution images. Again, the fractional contribution density histograms offered an alternative presentation method to the images of fractional contribution contrast. While the spatial contrast was lost in the histograms, the shape and peak position allowed a quick overview analysis of the trends in the data. Furthermore, histograms or phasor plots, which reduced the dimensionality of the data, could enable automated sample classification and/or image segmentation [21, 22].

## Abbreviations

CAD: Computer-aided design
CMOS: Complementary metal-oxide semiconductor
FLIM: Fluorescence lifetime imaging
FWHM: Full width at half maximum
IRF: Instrument response function
lidar: Light detection and ranging
MDF: Medium density fibreboard
PVA: Polyvinyl acetate
SPAD: Single-photon avalanche diode
TCSPC: Time-correlated single photon counting
TDC: Time-to-digital converter
UV: Ultraviolet
